# Prevalence of Aphthous Stomatitis: A Cross-Sectional Epidemiological Study

**DOI:** 10.7759/cureus.49288

**Published:** 2023-11-23

**Authors:** Shweta Mary Reddy, Jayanth Kumar Vadivel, Karthikeyan Ramalingam

**Affiliations:** 1 Oral Medicine and Radiology, Saveetha Dental College and Hospitals, Saveetha Institute of Medical and Technical Sciences, Saveetha University, Chennai, IND; 2 Oral Pathology and Microbiology, Saveetha Dental College and Hospitals, Saveetha Institute of Medical and Technical Sciences, Saveetha University, Chennai, IND

**Keywords:** herpetiform aphthous ulcers, major aphthous ulcers, minor aphthous ulcers, epidemiology and biostatistics, recurrent aphthous stomatitis

## Abstract

Introduction

Recurrent aphthous stomatitis (RAS) is a common ulcer of idiopathic etiology but is recurrent, causing painful ulcers in the non-keratinized mucosa. The disease is commonly seen in people aged 10-40 years. The etiology of RAS has yet to be well established, but several risk factors that lead to the development of RAS have been proven in the literature. With an unknown etiology, developing a definitive cure and maintaining disease remission remains challenging. An epidemiological survey will shed some light on the disease's prevalence, which could provide insights into disease management. This study aimed to study the prevalence of aphthous stomatitis among the patients visiting the dental outpatient services of a dental college in South India. The objectives were to discover the different subtypes of aphthous stomatitis and the age predominance of the type of ulcer.

Materials and methods

The data was collected from the case records of Saveetha Dental College and Hospitals, Chennai, India, dated from June 2019 to June 2023. The age and gender of the patients with RAS were recorded. The details collected were systematically arranged in an Excel sheet (Microsoft Corporation, Redmond, Washington, United States) and further analyzed using IBM SPSS Statistics for Windows, Version 24.0 (Released 2016; IBM Corp., Armonk, New York, United States) and chi-square tests were run to check for statistical significance.

Results

A total of 1,44,056 patients visited the Dental OPD during the study period. Of these, 1115 patients had RAS. When the data was analyzed, there was a three-fold increase in the occurrence of RAS during the coronavirus disease 2019 (COVID-19) pandemic, which was statistically significant (p=0.043). The most commonly affected age group was 26-40 years. Among the clinical variants of RAS, it was found that 82.5% of RAS patients had a minor variant of aphthous stomatitis.

Conclusion

This study shows the prevalence of different types of aphthous stomatitis, wherein we noticed that the minor clinical variant was the most common, followed by the major variant. The lesions were more commonly seen in women. However, the age group most commonly affected was 26-40 years.

## Introduction

The definition of health has been expanded to include optimum oral health. Optimum oral health is not just maintaining a healthy dentition but also maintaining good mucosal health. Daily activities such as eating, conversing, smiling, and contributing creatively to society influence a person's well-being. It is generally recognized that dental health is essential to overall wellness [1,2]. For general practitioners, the oral cavity is a lesser-known area, and only practitioners with special interest gain a brief overview of the common mucosal lesions. People frequently complain of pain, swelling, or growth in their mouth, but they do nothing to alleviate it. The diagnosis of oral lesions is critical in the field of dentistry. A correct diagnosis is essential since it can damage a patient's dental health and signal problems with their overall health [3].

One of the most common ulcers of the oral cavity is aphthous ulcers. Though the exact etiology is unknown, several risk factors exist, such as food allergens, genetic factors, and micronutrient deficiency. The disease is most commonly seen in women in the age group of 20-40 years. The word "aptha" or “aphthae” (plural) meaning small ulcers is derived from the Greek word "aphthi", which means fire. Since the ulcer tends to recur frequently, it is also called recurrent aphthous stomatitis (RAS). About 25% of people worldwide suffer from RAS [3,4]. The etiology of these lesions is unknown [5], although there are several risk factors proposed, ranging from trauma to intestinal diseases [6]. Several treatment options have been used to manage RAS with corticosteroids considered the first line of treatment [7]. Despite the modern therapies available, there is no drug to prevent the recurrence of the lesion in the high-risk group [8].

RAS can manifest clinically as minor, major, and herpetiform variants. Among the types, the most common presentation is minor aphthous ulcers, which have a 70-85% prevalence. The minor aphthous ulcer is characterized by circular, oval, or elongated lesions with a crateriform base measuring less than 1 cm and covered by a white-grey pseudomembrane and resolves in 10-14 days. The minor ulcers heal without scarring. The next common variant is the major aphthous ulcers. They tend to occur in singular numbers and measure greater than 1 cm in size. They take a longer time to heal, taking about 7-10 days, and leave behind a scar. Herpetiform ulcers are numerous pin-head-sized ulcers that occur in crops and heal without scarring. The ulcers present with burning, itching, or stinging sensation. The pain is severe during the first few days when the ulcer is forming and lessens as the ulcer cures. Speaking and eating may be affected by tongue lesions, which can be painful. Swallowing is uncomfortable due to the soft palate and throat sores [9]. Studies have revealed that people with poor oral hygiene and micronutrient deficiency are more likely to develop aphthous ulcers [10].

In this background, we planned a demographic study to assess the prevalence of RAS in patients visiting the dental OPD. The study aimed to determine the prevalence of different variants of RAS with the objective of studying the occurrence of RAS in the other genders. 

## Materials and methods

The study was conducted using the patient records of Saveetha Dental College and Hospitals, Chennai, India, from June 2019 to June 2023. The Institutional Ethics Committee of Saveetha Dental College and Hospitals approved the study (approval number: IHEC/SDC/UG-2171/22/OMED/222). Age and gender were the independent variables and the clinical type of aphthous was the dependent variable. Patients who had reported aphthous stomatitis were marked in the digital case sheets during the aforementioned period. The inclusion criteria and exclusion criteria are mentioned in Table 1. 

**Table 1:** Inclusion and exclusion criteria

Inclusion Criteria	Exclusion Criteria
Ulcers of idiopathic origin	Patients in whom a source of trauma or irritation was seen in the vicinity of the ulcer.
Ulcers of size < 1 cm were classified as minor	2. Ulcers that presented with rolled or everted margins.
Ulcers of size > 1 cm in size that persisted for > 2 weeks were classified as major	3. Ulcers in patients with systemic gastrointestinal diseases.
Ulcers occurring in crops were classified as herpetiform	4. Ulcers noticed in pregnant mothers.

The fact that the data was gathered from a verified and standardized database allowed for establishing the study's internal validity. The external validity is confirmed because the data comes from a reproducible clinical scenario. The data was finally tabulated in a Microsoft Excel sheet (Microsoft Corporation, Redmond, Washington, United States) once the appropriate data about aphthous stomatitis had been gathered. IBM SPSS Statistics for Windows, Version 24.0 (Released 2016; IBM Corp., Armonk, New York, United States) was used for data analysis. The normality of the data was checked with Kolmogorov-Smirnov and Shapiro-Wilks tests. The data was analyzed to see if there was any association between age, gender, and the different clinical manifestations of aphthous stomatitis.

## Results

A total of 1,44,056 patients visited the Dental OPD from June 2019 to June 2023. Among them, 1115 patients were diagnosed with RAS, which included 737 males and 378 females. The data of patients visiting the dental hospital during the study period and the number of RAS recorded yearly are given in Table 2. It can be seen that though there has been a fall in the number of patients visiting the Dental OP during the coronavirus disease 2019 (COVID-19) lockdown, there has been a spike in the number of RAS cases. The results, when compared statistically, show a significant difference (P=0.043).

**Table 2:** Comparison between the total number of patients and patients with RAS RAS: recurrent aphthous stomatitis

Year	Total patients	Patients with RAS, frequency	Patients with RAS, percentage
2019 from June	24,635	81	0.33%
2020	22,235	404	1.82%
2021	32,786	421	1.28%
2022	44,875	145	0.32%
2023 till June	19,525	64	0.33%
Total	1,44,056	1,115	0.78%

**Figure 1:**
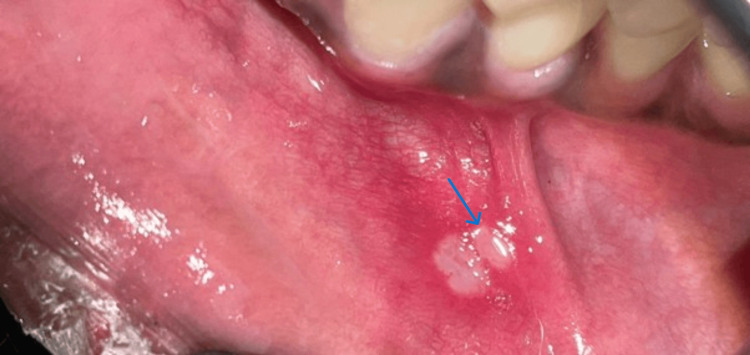
Aphthous stomatitis minor in the lower labial mucosa (indicated by the arrow)

**Figure 2:**
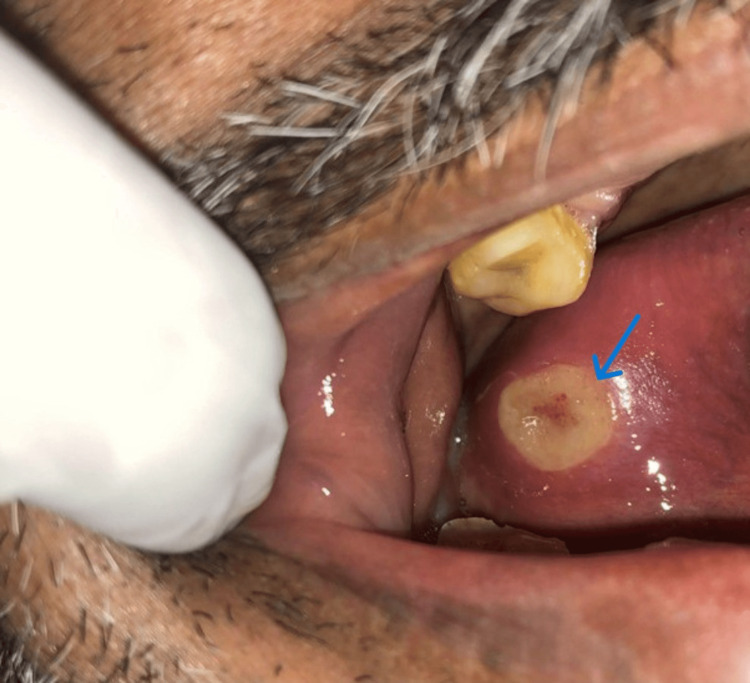
Aphthous stomatitis major in the lateral border of the tongue (indicated by the arrow)

**Figure 3:**
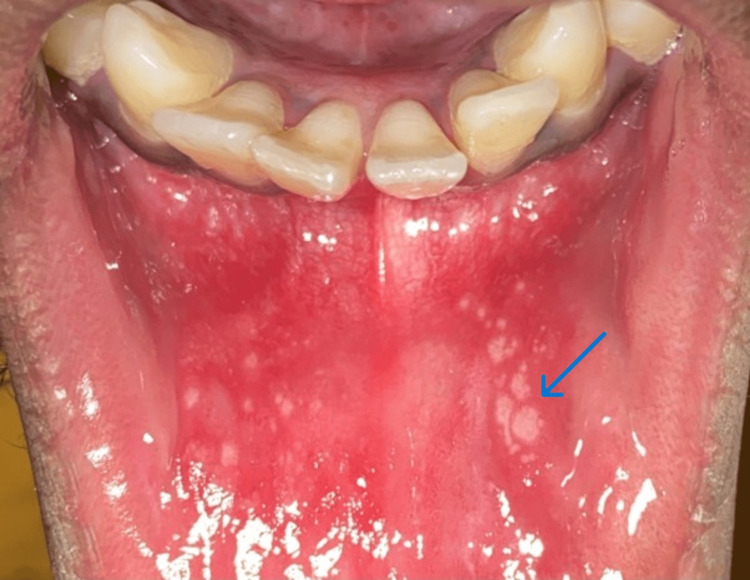
Herpetiform aphthae in the lower labial mucosa (indicated by the arrow)

Among the clinical presentations of aphthous, the most commonly noted was the minor aphthous (Figure 1), followed by the major aphthous (Figure 2), and the herpetiform variant was the least common (Figure 3). A total of 1027 (92.11%) patients presented with minor aphthous stomatitis. The most common age groups of presentation were 26-40 years and < 25 years. This was followed by major aphthous ulcers in 72 (6.45%) patients. Unlike the minor variant, the major variant was commonly seen in the age group of < 25 years. The third variant was the herpetiform aphthae, which was seen in 15 (1.34%). A statistical analysis was done using a chi-square test, which showed the predominance of the types of ulcers across the age groups had a significant difference (P<0.05). 

**Figure 4:**
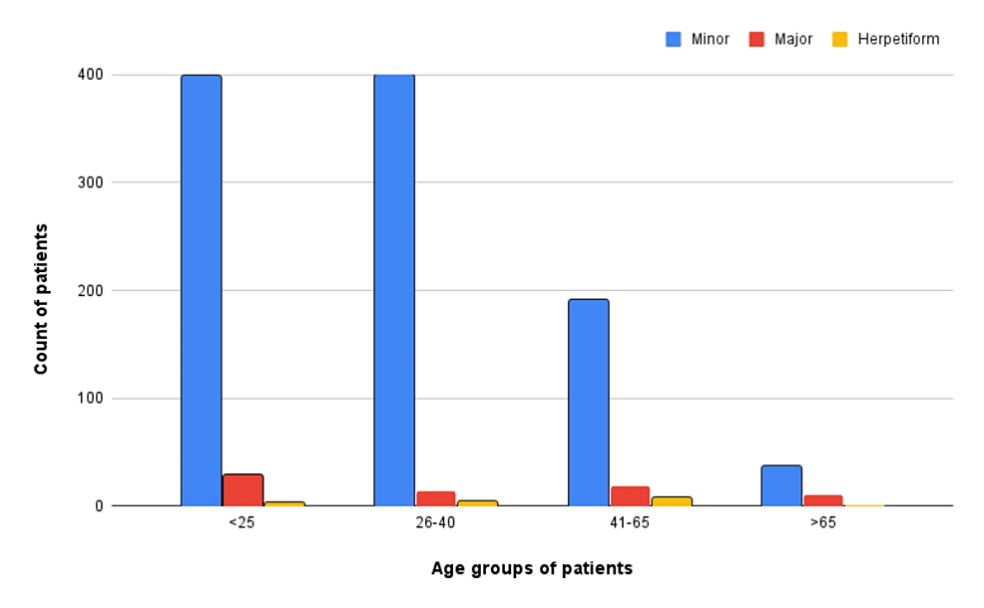
Various clinical variants in aphthous stomatitis according to different age categories; the most common clinical variant was minor aphthous (1027 cases), followed by major (72 cases), and then herpetiform (16 cases).

The association between the age group and the clinical variant showed that the majority of the aphthous minor cases were recorded in the age groups of < 25 years and 25-40 years. The data was compared by Pearson correlation with a post hoc test and were statistically significant (p=0.024) (Figure 4).

**Figure 5:**
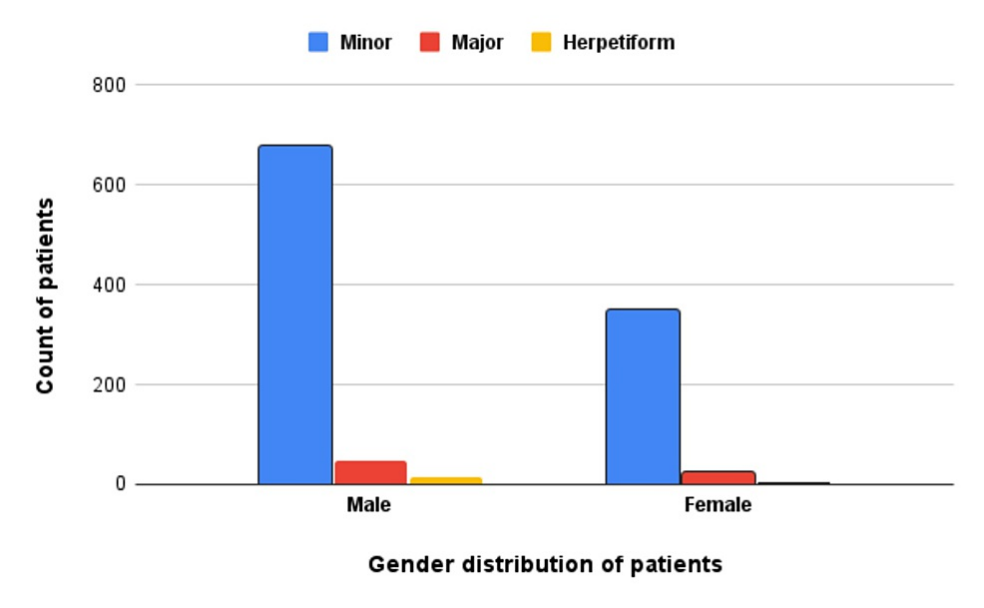
Clinical variants of aphthous stomatitis according to gender; minor clinical variant was the most prevalent type of aphthous stomatitis, observed more in males than females.

With regard to gender, 736 (66.01%) males were affected compared to 379 (33.99%) females. The difference was also statistically significant (p=0.03) (Figure 5).

## Discussion

The most painful ulcerative disorder affecting the oral cavity is RAS. The level of pain and discomfort is significantly greater than the size of the ulcer, according to pain measurement methods such as the visual analog scale and numerical rating scale. Despite several advancements, there still exists a lacuna in identifying the etiology of the ulcers. However, there are several reports that state that stress is a common risk factor for the development of aphthous ulcers, usually of the minor variant. The role of stress leads to cytokine dysregulation, which could increase susceptibility to mucosal breakdown [11]. During a COVID-19 study, it was found that there was an increase in the odds ratio of the occurrence of RAS [12]. In the present study, it was found that there was a three-fold increase in the number of RAS cases during the COVID-19 pandemic period despite a fall in the outpatient inflow during the same period.

In a survey study done by Verma et al., wherein a pre-tested questionnaire was circulated among 1,134 dental students from North East India, it was found that 371 (32.7%) students reported the occurrence of RAS within six months [13]. In this subgroup, only 135 students had a higher score on the perceived stress scale; the rest of them did not report significant stress scores. This was a study carried out during the pandemic period and there was a 3.2-fold increase in the occurrence of the ulcers. The findings of this study are in line with the observations of the current study, so apart from stress there has been some other factor during the pandemic period which led to a surge in RAS. In a case series reported by Wu et al., there were several asymptomatic COVID-19 patients who were reverse transcriptase-polymerase chain reaction (RT-PCR) positive for COVID-19 reporting with RAS [14]. So the increase in RAS during the pandemic period might probably have been asymptomatic COVID-19 cases.

The age of occurrence of this lesion is predominantly in the young to the middle age. In a Swedish study, it was found that the majority of the cases were recorded in the 15-40 years age group [15].** **The results of the current study are also in line with the observations of the Swedish study, with 848 (82.5%) of the RAS cases in our study occurring in the < 40-year age group. The occurrence in the young to middle age may be probably due to the association between stress and RAS. Some papers show a genetic susceptibility with familial history to RAS. In a study by Al Omiri et al., they found that there was an occurrence of RAS in some family members [16]. Furthermore, they also studied the stress patterns of the participants and found that, based on the Neuroticism-Extraversion-Openness Five-Factor Inventory, the scores were higher in certain families [16]. From this, we can conclude that though the etiology of RAS is not clearly understood, stress plays a significant role in the causation of these ulcers. Also, genetic factors play a role in the way people respond to stress, which in turn may lead to the causation of RAS.

Earlier studies have shown that there is a female preponderance of the lesion. According to the research done by Safadi et al., women were most commonly affected, and 80% of RAS was of the minor variant [17]. In our study, it was found that the minor variant was the most common ulcer; however, in our research, it was found that the ulcers were most commonly seen in men. This difference could be due to the sample ethnicity variation between the studied groups. Similar to the current study, a study by Kaur et al. also showed a male predominance of the lesions [18]. The study by Verma et al. too showed that more cases of RAS have been reported in males during the pandemic period [13]. Based on the variant findings, we conclude that gender cannot be used as a risk factor predictor for the occurrence of RAS.

In a study by Gurkan et al., it was found that aphthous stomatitis tended to occur more often in childhood [19]. In a subgroup of the cases recorded by them in the <18-year age group, it was found that the mean age of occurrence was 9.6 years. Furthermore, they also stated that in this age group, there was a frequent lesion recurrence, with the average length of illness being 3.6 years. They also said minor aphthous stomatitis was the most common clinical variant [19]. The probable reason for this could be the nutritional deficiencies in the younger age group since the sample was from a developing country. However, in the same paper by Gurkan et al., it was found that in the adult population, the most common age group to be affected was 26-40 years [19]. In the current study also, it was found that aphthous stomatitis was common in people under 40, with the most common variant being aphthous minor ulcer. The study by Gurkan et al. [19] was done in the North Indian population, and similar data is reflected in our study, which was done in South India. This shows that the demographics of the lesion are the same in both parts of India.

Limitation

The primary limitation of the study was that it was unicentric, and only one ethnic group was studied. Also, the patients' follow-ups for the recurrence of the lesion were not recorded.

## Conclusions

Within the study's limitations, it can be concluded that the minor variant of RAS was the most common type, and the disease was commonly seen in the young to middle-aged group of patients, which is in line with the global prevalence of the disease. However, in the present study, it was seen that men are more commonly affected than women, which could be due to the genetic factors found in this ethnic group of patients. There is mixed data from the global population to support this statement. However, one major observation was that there was a threefold increase in lesions during the pandemic period, which could probably be due to the increased stress levels of individuals during the pandemic period. Thus, the occurrence of RAS seems more so in younger individuals and it can be said that the lesions may be associated with stress.
